# Patients with schizophrenia show aberrant patterns of basal ganglia activation: Evidence from ALE meta-analysis

**DOI:** 10.1016/j.nicl.2017.01.034

**Published:** 2017-02-11

**Authors:** Jessica A. Bernard, Courtney E. Russell, Raeana E. Newberry, James R.M. Goen, Vijay A. Mittal

**Affiliations:** a Department of Psychology, Texas A&M University, United States; b Texas A&M Institute for Neuroscience, Texas A&M University, United States; c Department of Psychology & Neuroscience, University of Colorado Boulder, United States; d Department of Psychology, Northwestern University, United States; e Department of Psychiatry, Northwestern University, United States; f Institute for Policy Research, Northwestern University, United States; g Department of Medical Social Sciences, Northwestern University, United States

**Keywords:** Basal ganglia, Schizophrenia, Meta-analysis, Dopamine hypothesis, Neuroimaging

## Abstract

The diverse circuits and functional contributions of the basal ganglia, coupled with known differences in dopaminergic function in patients with schizophrenia, suggest they may be an important contributor to the etiology of the hallmark symptoms and cognitive dysfunction experienced by these patients. Using activation-likelihood-estimation meta-analysis of functional imaging research, we investigated differences in activation patterns in the basal ganglia in patients with schizophrenia, relative to healthy controls across task domains. This analysis included 42 functional neuroimaging studies, representing a variety of behavioral domains that have been linked to basal ganglia function in prior work. We provide important new information about the functional activation patterns and functional topography of the basal ganglia for different task domains in healthy controls. Crucially however, we demonstrate that across task domains, patients with schizophrenia show markedly decreased activation in the basal ganglia relative to healthy controls. Our results provide further support for basal ganglia dysfunction in patients with schizophrenia, and the broad dysfunction across task domains may contribute to the symptoms and cognitive deficits associated with schizophrenia.

## Highlights

• The basal ganglia are connected to the cortex through multiple parallel circuits.
• Basal ganglia function has been implicated in patients with schizophrenia.
• Meta-analysis demonstrated a functional activation topography in the basal ganglia.
• Patients with schizophrenia show a decrease in basal ganglia activity across studies.
• These are important for our understanding of symptoms in this clinical population.

## 1 Introduction

Across the psychosis spectrum, from youth exhibiting risk syndromes to patients with schizophrenia, there is evidence for basal ganglia abnormalities, including functional and structural differences, as well as dopaminergic receptor differences (Ellison-Wright et al., 2008, Fusar-Poli et al., 2011, Howes et al., 2011a, Howes et al., 2011b, Perez-Costas et al., 2010, Salvador et al., 2010, Yoon et al., 2013). Leading etiological theories of psychosis have implicated the basal ganglia and fronto-striatal dysfunction (Graybiel, 1997, Robbins, 1990). Indeed, the third and most recent version of the dopamine hypothesis purports that numerous factors including genetic, environmental, and psychosocial factors, converge to result in dopaminergic dysfunction, and a final common pathway rests at the level of presynaptic striatal dopaminergic control (Howes and Kapur, 2009). Given that the basal ganglia and their function are highly reliant upon a healthy dopaminergic system, as evidenced by diseases such as Parkinson's and Huntington's (e.g., Bernheimer et al., 1973, DeLong, 1990, Obeso et al., 2000), and the purported role of dopamine in psychosis and schizophrenia, these brain areas are of particular interest as we try to better understand the heterogeneous signs and symptoms experienced by patients with schizophrenia.

Connections between the basal ganglia and cortex are subserved by parallel circuits connecting distinct cortical regions with distinct regions of the basal ganglia. There are four primary cortico-striatal-pallido-thalamic circuits—the motor, occulomotor, limbic, and prefrontal circuits—defined based on their cortical projections (Alexander and Crutcher, 1990, Alexander et al., 1986, Hoover and Strick, 1993, Middleton and Strick, 2002, Middleton and Strick, 2000a, Middleton and Strick, 2000b). Based on the respective cortical projections, each regulates distinct behaviors and functions including motor behavior, reward processing, emotion, and higher-order cognitive processes such as language, working memory, and executive function (Tekin and Cummings, 2002). These circuits are organized in direct and indirect pathways linked by excitatory glutamatergic and inhibitory GABA-ergic projections. However, each passes through the basal ganglia, which are richly innervated with dopamine (DA), that balances the direct and indirect pathways through differential actions of DA on striatal D1 and D2 receptors (DeLong and Wichmann, 2007, Obeso et al., 2014a, Obeso et al., 2014b). Thus DA receptor, or broader circuit abnormalities may affect those tasks regulated by basal ganglia structures, as well as a host of apparently disparate functions characterized in schizophrenia (Mittal et al., 2010, Robbins, 1990). Indeed, in a recent review, Dandash et al. (in press) suggested that the circuit linking the dorsal striatum to the lateral prefrontal cortex may be especially important in the pathophysiology of psychosis, as dysfunction is seen across the spectrum of risk and disease.

While early work was conducted using animal models, the parallel loops of the basal ganglia have been demonstrated in the human brain as well using diffusion tensor imaging (Draganski et al., 2008), resting state functional connectivity (Di Martino et al., 2008a), and with meta-analytic investigations of co-activation patterns (Postuma and Dagher, 2006). Indeed, the work of Draganski et al. (2008) demonstrated a connectivity gradient with the cortex that follows a rostral to caudal pattern across structures (caudate, putamen, pallidum). The connectivity profiles of the most rostral areas are associated with the orbital and medial prefrontal cortex and move caudally to the prefrontal, premotor, and motor cortices. The diffuse cortical connections, in conjunction with behavioral evidence in patient populations suggesting that dopaminergic and basal ganglia dysfunction result in motor, cognitive, and affective deficits (Jurgens et al., 2008, Middleton and Strick, 2000a, Middleton and Strick, 2000b, Tekin and Cummings, 2002), indicate that the basal ganglia are crucial for performance across domains. Furthermore, this suggests that within each nucleus, processing occurs on a variety of task information, due to the differing input and output loops with the cerebral cortex.

The wide array of behavioral contributions of the basal ganglia and purported role of dopamine in psychosis make an improved understanding of basal ganglia function in schizophrenia of particular interest. As noted, structural differences in basal ganglia volume are seen across the psychosis spectrum. Smaller volume in basal ganglia nuclei is seen in generally healthy individuals who report psychotic-like experiences (non-clinical psychosis; Mittal et al., 2013), individuals with schizotypal personality disorder (Levitt et al., 2002), those at clinical high risk for psychosis (CHR; i.e., exhibiting prodromal syndromes; Mittal et al., 2010), and finally, in those with a diagnosis of schizophrenia (e.g., Corson et al., 1999). In addition to these structural differences, functional connectivity patterns of the basal ganglia are also altered in CHR individuals (Dandash et al., 2014), as well as in patients with schizophrenia (Khadka et al., 2013, Salvador et al., 2010, Tu et al., 2012). These findings suggest a general decrease in resting state connectivity of the basal ganglia in key cortical networks, though there is some evidence for increases in at-risk individuals, and when basal ganglia connectivity is investigated in conjunction with the default mode network (Dandash et al., 2014, Salvador et al., 2010). However, there has also been an increase in the number of investigations completing task-based functional imaging (fMRI) in schizophrenia, that stand to provide further insight into the basal ganglia and basal ganglia function in this important patient population. Synthesizing across these studies to look at basal ganglia activation patterns stands to provide critical new insights into our understanding of the basal ganglia in schizophrenia.

Here, we conducted an activation likelihood estimation (ALE) meta-analysis, which produces an estimation of the likelihood that a given voxel, when considering all the voxels in the brain, is activated during the performance of a particular task (Eickhoff et al., 2009). We focused on specific tasks that align with basal ganglia circuits to address two key questions. First, what is the functional topography of task-based activation in the basal ganglia in healthy adults? While both diffusion tensor and resting state imaging have provided us with an excellent understanding of basal ganglia networks in the human brain (Di Martino et al., 2008b; Draganski et al., 2008), and though there is a vast fMRI literature, to our knowledge the functional topography of the basal ganglia has not been mapped in healthy adults with meta-analytic methods. Furthermore, understanding basal ganglia organization in healthy controls is important for work in schizophrenia. Though our methodologies are optimized for investigating patients with schizophrenia, our analyses of the control participants allow for important new insights as to the functional topography of the basal ganglia based on activation patterns across task domains. With respect to the functional topography in healthy controls, we expected to see a gradient of activation foci across studies in patterns consistent with the rostral to caudal connectivity gradient demonstrated by Draganski et al. (2008) within individual basal ganglia nuclei. As such, we expected to see some degree of motor and cognitive activation across all the nuclei in the basal ganglia, but in an organized manner. The most rostral regions of the caudate and putamen would be associated with higher cognitive processing, while the most caudal would be associated with motor tasks. With respect to reward, we also expected to see significant activation overlap in the ventral striatum. Second, we sought to understand differences in basal ganglia activation and functional topography in individuals with schizophrenia. How do these activation patterns differ across task domains in the presence of this disorder? While the dopamine hypothesis (Howes and Kapur, 2009) and hyperkinetic movements seen in CHR and schizophrenia spectrum populations indicate an excess of dopamine in the basal ganglia in patients with schizophrenia (Howes et al., 2011a, Howes et al., 2011b, Mittal et al., 2008, Pappa and Dazzan, 2009), functional connectivity of the basal ganglia seems to be decreased. Given these functional connectivity decreases we predicted that the patient group would show decreased activation across task domains when compared to controls. Furthermore, this would be consistent with our recent meta-analytic work investigating the cerebellum, where we also saw decreased activation across studies, relative to controls (Bernard and Mittal, 2015). However, we did not expect to see alterations in the topography of activation, and as such we expected that the predicted rostral-caudal gradient of activation foci would remain the same.

## 2 Method

### 2.1 Literature search

Articles for inclusion were identified using two independent PubMed literature searches (http://www.ncbi.nlm.nih.gov/pubmed). For both searches, we used identical sets of search limits which included “Adult 19–44”, “English”, and “Humans”. Our meta-analysis was limited to this adult group so as to minimize the potential confounding effects of advanced age with disease state, as the basal ganglia are impacted by aging and there are age-related decreases in dopamine (reviewed in Seidler et al., 2010). Additional exclusion criteria for studies were consistent with our previous functional neuroimaging meta-analyses (Bernard and Mittal, 2015, Bernard and Seidler, 2013). That is, we excluded articles that did not use functional neuroimaging methods, did not report coordinates in standard space, studies using only region of interest analyses, and studies that did not use standard contrast analysis (e.g., independent components analysis). In addition, for the purposes of our investigation of the basal ganglia here, we limited our analyses to studies that reported activation in the basal ganglia, after completing two broad searches of the literature. However, it is notable that we included activations that were described as “midbrain” as this is the location of the substantia nigra, a component of the basal ganglia. With current imaging methods, specifically locating the substantia nigra can be particularly challenging, and as such we were more liberal in our inclusion of midbrain activation in hopes of encompassing this region. Also of note, the activation could be in either patients or controls. That is, studies were included even if activation was only limited to one group.

Search terms were defined so as to encompass the neuroimaging literature broadly, and to parallel the strategy used in our recent meta-analysis of the cerebellum in psychosis (Bernard and Mittal, 2015). All analyses and papers included were those available as of May 15, 2015. The search “schizophrenia AND neuroimaging” returned 2424 articles, while our second search of “basal gangl* AND schizophrenia AND imaging” returned 258 papers. The first search was designed to broadly encompass all of the available neuroimaging literature on patients with schizophrenia. This allowed us to cast a wide net. Our second search was more focused on the basal ganglia in order to make sure that we included as many studies as possible showing basal ganglia activation in studies of schizophrenia (either in controls or patients). As described above, we limited our analyses only to those studies including activation in the basal ganglia in one group. First, this is critical for our ability to investigate group differences in basal ganglia activation across studies, as a meta-analysis of over 2500 manuscripts, of which only a subset included activation of interest, would actually limit our power to detect group differences. We are unable to detect differences if there is no activation present in the first place. Second, many of the studies that were the result of this search included tasks where one might not expect any activation in the basal ganglia. As such, we limited our inclusion sample, to only look at group differences in investigations where basal ganglia activation was present.

As indicated, because the basal ganglia are known to have multiple loops and connections with the cortex in prefrontal, motor, and limbic regions (Draganski et al., 2008), we focused on task domains associated with these distinct loops that are also known to be impacted in patients with schizophrenia. Thus, we included functional studies where the task used fell into one of the following categories: motor function, executive function/attention, working memory, emotional processing, language, and reward processing (Cohen and Minor, 2010, Fioravanti et al., 2005, Lee and Park, 2005, Mesholam-Gately et al., 2009, Obeso et al., 2014a, Pappa and Dazzan, 2009, Strauss et al., 2014). Notably however, because we were investigating a clinical population, the tasks were designed to optimize performance in both groups. As such, it may be the case that we are underestimating the activation in the healthy control groups due to task difficulty and other related factors. Furthermore, we did not take into account task performance as our primary interest was based on functional activation patterns, and this was not an exclusion criteria in our selection of papers for analysis.

Papers for inclusion were narrowed down by first excluding those that did not use functional imaging. Then, the remaining papers with functional analyses were investigated to determine whether or not they used standard general linear model analyses, and included activation foci in the basal ganglia presented in standard space (either Montreal Neurological Institute (MNI) or Talairach). Finally, all control data come from papers that included both controls and patients with schizophrenia, though in several instances papers that only investigated patients with schizophrenia were included. After thoroughly examining all papers returned in our PubMed searches to ensure that they met the inclusion criteria for our study, we ended up with 42 studies (concatenated across all domains) for inclusion in our analyses, and this resulted in data from a total of 707 patients with schizophrenia and 583 controls (concatenated across all 42 studies). The included articles as well as description of the tasks included and the neuroimaging contrasts investigated are presented in Table 1.

**Table 1:** Study information for all papers included in the meta-analysis, including imaging modality, sample size, a general task description and the number of foci per group. Each general task domain is grouped separately.

Study	Imaging modality	N, SCZ	N, CON	Task	# SCZ foci	# CON foci
Emotion						
Lakis et al., 2011	3 T MRI	37	37	Retrieval of high arousal emotional images relative to neutral images	0	1
Taylor et al., 2011	3 T MRI	21	21	Preference (yes or no to the prompt “Like”) in emotional faces (positive, negative, fear) contrasted with gender discrimination condition	0	1
Takahashi et al., 2004	1.5 MRI	15	15	Participants gave ratings of neutral, unpleasant, and pleasant IAPS pictures	0	1
Kumari et al., 2011	1.5 MRI	56	0	Emotional faces & participants indicated gender. Neutral relative to a control oval included here only at baseline assessment	1	n/a
Taylor et al., 2005	PET	18	10	Rating of emotional images (IAPS); contrasted emotional and non-aversive images	0	2
Phillips et al., 1999	1.5 T MRI	10	5	Implicit facial emotion processing during a gender discrimination task; Patient group included 5 paranoid and 5 non-paranoid individuals	0	5
Bourque et al., 2013	3 T MRI	14	21	Retrieval of positive relative to neutral IAPS pictures	0	2
Johnston et al., 2005	1.5 T MRI	11	15	Gender and emotion discrimination of emotional faces, relative to shapes	0	2

Executive function/attention						
Zandbelt et al., 2011	3 T MRI	24	24	Inhibitory control as measured using a stop-signal anticipation task. Included analyses of stop-signal probability parametric effects and successful and failed stop trials.	9	18
Liddle et al., 2006	1.5 T MRI	28	28	Selective attention, auditory oddball; targets relative to baseline and novel stimuli	0	4
Royer et al., 2009	1.5 T MRI	19	12	Hayling sentence completion task with completion using the expected word, or an unrelated word (inhibition condition)	0	2
Camchong et al., 2005	1.5 T MRI	14	14	Occulomotor delayed response task with saccades to peripheral locations	0	1
Tu et al., 2006	3 T MRI	10	10	Antisaccade task	0	2
Harrison et al., 2007	3 T MRI	16	14	Multi-source interference task with three numbers with congruent and incongruent blocks	0	1
Ojeda et al., 2002	PET	11	10	Sustained attention, mental counting with auditory stimulation	0	2

Language						
Mashal et al., 2013	3 T MRI	14	14	Figurative language task made up of 96 pairs of words with literal or metaphoric associations, or no relationship. Processing across relation levels compared	3	3
John et al., 2011	3 T MRI	24	24	Word generation and word repetition	0	13
Ragland et al., 2008	3 T MRI	14	13	Semantic word generation	3	2
Stephane et al., 2006	PET	18	12	Word reading (nouns), relative to looking at nouns	1	0
Weinstein et al., 2006	1.5 T MRI	12	11	Listening to speech (English, Mandarin, and reversed English)	2	0

Motor						
Horan et al., 2014	3 T MRI	23	23	Imitation, observation, and execution of finger and face movements	7	8
Müller and Klein, 2000	1.5 T MRI	3	3	Self-paced finger tapping	2	2
Marvel et al., 2007	PET	12	11	Implicit sequence learning (serial reaction time task) and random button presses	0	3
Müller et al., 2002	1.5 T MRI	10	10	Unilateral, finger-to-thumb opposition task (sequential movement); Patients divided into 3 groups of 10 based on medication. Here only the untreated group showed activation, so only 10 participants are represented	1	2
Kumari et al., 2002	1.5 T MRI	6	6	Implicit sequence learning	0	2
Mattay et al., 1997	1.5 T MRI	8	8	Sequential finger-to-thumb opposition and individually created random movement sequence. Analysis completed on only 7 subjects as one outlier per group was removed	1	3

Reward						
Gradin et al., 2011	1.5 T MRI	15	17	Instrumental reward learning task using fractal pictures and water reward (fluid withdrawal the night before) given on a probabilistic schedule	2	4
Insel et al., 2014	1.5 T MRI	22	0	Reinforcement learning, with conditions for monetary gain, and avoiding monetary loss	8	n/a
Schlagenhauf et al., 2014	3 T MRI	24	24	Reversal learning task, two sessions of 100 trials with reward and punishment	0	7
Walter et al., 2009	3 T MRI	16	16	Monetary incentive task, parametric variation of wins	4	3
Gradin et al., 2013	1.5 T MRI	14	18	Pavlovian reward learning task with water reward after fluid withdrawal, using fractal pictures	0	2
Morris et al., 2012	3 T MRI	21	16	Reward prediction error during Pavlovian cue-outcome card game with both expected and unexpected rewards	0	12

Working memory						
Guse et al., 2013	3 T MRI	25	22	Verbal working memory, 2-back relative to 0-back. Data from controls in two groups of 11 and only include pre-TMS intervention	0	2
Luck et al., 2010	2 T fMRI	16	17	Verbal working memory Sternberg variant with incorrect lures	0	3
Royer et al., 2009	1.5 T MRI	19	12	N-back, 2-back relative to 0-back	2	0
Pae et al., 2008	1.5 T MRI	12	11	N-back (2-back)	1	2
Manoach et al., 2000	1.5 T MRI	9	9	Sternberg verbal working memory of digits at high (5) and low (2) memory load	2	0
Avsar et al., 2011	1.5 T MRI	10	8	Visual delayed match-to-sample task	0	1
Kircher et al., 2009	1.5 T MRI	14	0	N-back task, 2-back and 0-back in first-episode patients with genetic risk	1	n/a
Kim et al., 2003	PET	12	12	N-back task (2-back) with shapes, relative to control focused attention task	0	1
Johnson et al., 2006	3 T MRI	18	18	Sternberg verbal working memory task using letters, with medium and difficult load conditions	0	4
Yoo et al., 2005	1.5 T MRI	12	12	N-back task (2-back) with neutral faces	0	2

### 2.2 ALE meta-analysis

All analyses were conducted using BrainMap GingerALE 2.3.5 (http://brainmap.org; Eickhoff et al., 2009, Eickhoff et al., 2012, Turkeltaub et al., 2012), using the most recent algorithm designed to minimize the impact of individual experiments (Turkeltaub et al., 2012). The foci were first concatenated together for analysis across all domains, and then were organized by specific task domain. In both cases, the foci were divided into those associated with patients and controls. Because there are two standard atlas spaces (MNI and Talairach) it is essential to ensure that all foci across studies are in the same space so as to more accurately compare the spatial locations of the activation foci. Thus, we converted all coordinates in Talairach space to MNI space. For all studies where the data were normalized directly to Talairach space, and for those that specified the use of the Lancaster transform (icbm2tal), we transformed them to MNI space using the Lancaster transform (Lancaster et al., 2007). We also used this approach for articles published after the Lancaster transform was made available, but for which there was no transform algorithm specified in the manuscript text. For articles where the Brett transform (mni2tal) was used to bring MNI data into Talairach space, and for those articles published prior to 2007 and there was no transform specified, we used the inverse Brett transform to bring the foci back to MNI space. All transformations were completed using tools available in GingerALE.

Activation foci in MNI space were organized into text files, which were then entered into GingerALE. The software algorithm computes ALE values for all voxels in the brain, and produces an estimation of the likelihood that a given voxel is activated during the performance of a particular task (Eickhoff et al., 2009). As part of the analysis in GingerALE, a full-width half maximum (FWHM) Gaussian blur is used on each set of foci, though the size of the blur is adjusted automatically based on the number of subjects contributing to each respective set of foci (Eickhoff et al., 2009). That is, the meta-analysis procedure takes into account the number of subjects in a particular study automatically. Thus, while the studies included here ranged greatly in the number of participants in both the patient and control groups, the analysis procedure itself accounted for this. In our analyses, the output indicated that the FWHM blur ranged from 8.7 to 12.97 mm, across all analyses. All analyses were conducted using the smaller more conservative mask option available in GingerALE. Within-group analyses for each task (and in the combined analysis pooling across all tasks) were evaluated using cluster-level correction, consistent with the recommendations of Eickhoff et al. (2012). All ALE maps were first thresholded using an uncorrected p < 0.001 as the cluster-forming threshold, and then with FDR p < 0.05 for cluster-level inference, with 5000 threshold permutations. Group contrasts (subtraction analyses) and conjunctions for each individual task were evaluated using an uncorrected p < 0.05 with 10,000 p-value permutations and a minimum cluster size of 50 mm^3^, while the pooled comparison had a minimum cluster size of 100 mm^3^. This approach was taken because GingerALE is not very robust when small numbers of studies (fewer than 15 per group) are used for group contrasts. However, this is consistent with the approach we took in our meta-analytic work investigating the cerebellum (Bernard and Mittal, 2014), and has also been used in other recent meta-analyses (e.g., Garrison et al., 2013, Stawarczyk and D'Argembeau, 2015). The cluster minima were implemented to account, at least in part, for the lack of additional statistical correction. While we are looking at small structures given our focus on the basal ganglia, we have chosen this more conservative cluster size in the interest of limiting false positives as effectively as possible. The resulting foci from all analyses were localized using the AAL atlas in MRICron, as well as the Harvard-Oxford subcortical atlas in FSL.

To investigate the functional topography of the basal ganglia in the control group only, we conducted contrast analyses comparing foci associated with a particular task domain (for example, “motor”) and we compared these foci with the combined foci for all other task domains (“everything except motor”). This allowed us to investigate whether there are any foci of activation for a particular task domain, distinct from the activation for all of the other task domains in question, which is crucial for investigating topographical functional organization across studies.

Contrast analyses comparing patients with schizophrenia and controls were computed using the foci from each group, as opposed to using group contrasts that were in some cases reported in the original studies included here. They represent a subtraction of the resulting ALE maps (or addition in the case of the conjunction analysis). Not all studies included healthy controls in conjunction with patients with schizophrenia. Therefore, to increase our power and include all possible investigations, we contrasted the foci between the groups on all of the behavioral domains of interest and pooled across tasks.

## 3 Results

### 3.1 Task activation overlap in controls

**Fig. 1:**
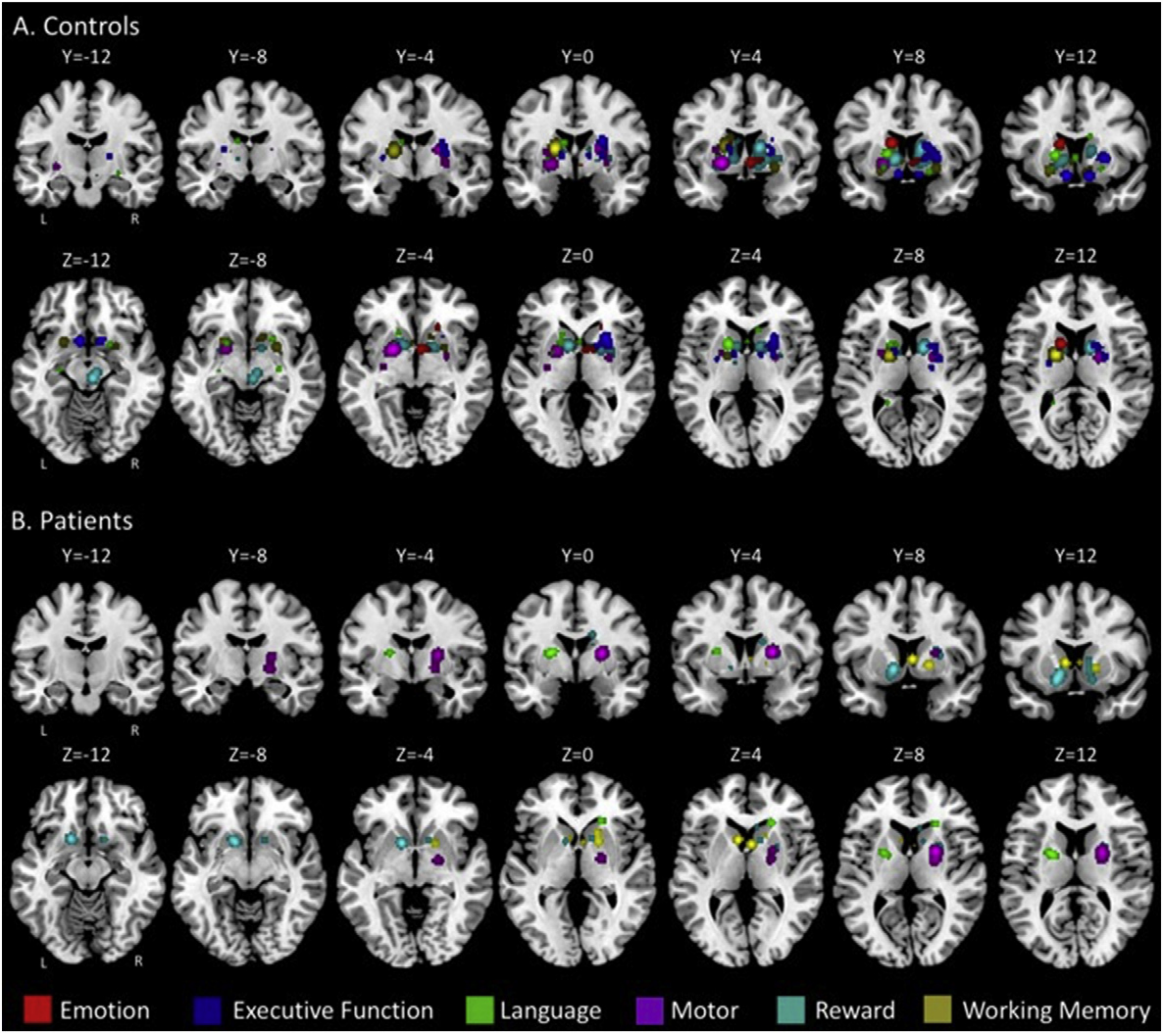
ALE result maps for each task domain presented for the controls (**A**) and the patients with schizophrenia (**B**). Both coronal and axial slices are presented. Notably, in the patients with schizophrenia there were no significant areas of functional activation overlap for the emotion and executive function/attention domains, and as such no clusters are pictured.

Overlap in activation across studies was first investigated for the control and patient groups separately. The control analysis was especially important for establishing normative patterns of activation and the functional topography of the basal ganglia in the healthy adult brain. While resting state and diffusion tensor parcellations have provided excellent maps of the circuitry of these regions (Draganski et al., 2008, Di Martino et al., 2008) that inform our interpretation of the functional data, the topography with respect to specific task domains, up to this point, had not been delineated to our knowledge. Table 2 presents detailed information about the peak coordinates, weighted centers, cluster sizes, and anatomical region of activation by group and task domain, while Fig. 1 shows the patterns of overlapping activation in each task domain, separated by group. General patterns will be summarized here.

**Table 2:** Significant clusters of overlap for each individual task domain separated by group. Results include the cluster size, the weighted center of the significant overlap, the local peaks within the cluster as well as the anatomical location within the basal ganglia of the activation overlap. Notably, there were no significant areas of activation overlap in the patient group for the emotion and executive function/attention task domains and as such no results are included.

Cluster	Cluster size (mm^3^)	Weighted center (x, y, z)	Local Extrema (x, y, z)	Location	ALE value (× 10^− 3^)
Emotion					
Controls					
Cluster 1	824	− 13.1, 9.4, 13.8	− 12, 10, 14	Caudate (body)	14.16
Cluster 2	752	7.2, 4.6, − 2.9	4, 4, − 4	Caudate (head)	10.60
Cluster 3	240	19.9, 24.5, − 3.4	20, 24, − 4	Caudate (HEAD)	7.26
Cluster 4	224	− 21, − 18, 18	− 21, − 18, 18	Thalamus	8.23
Cluster 5	224	18, − 18, 21	18, − 18, 21	Caudate (tail)	8.23

Patients					
N/A					

Executive function/attention					
Controls					
Cluster 1	3288	23.5, 6.7, 6.7	24, 16, 0	Putamen	13.45
			24, 0, 18	Putamen	12.08
			18, 8, 12	Caudate (body)	9.88
			24, 0, 4	Putamen	9.69
			28, − 4, 8	Putamen	9.67
			16, 8, 0	Putamen	9.22
Cluster 2	648	− 7.5, 11, − 13.8	− 8, 12, − 14	Sub-lobar Gray Matter	15.02
Cluster 3	544	11.8, 11.2, − 14.1	12, 12, − 14	Sub-lobar Gray Matter	13.04
Cluster 4	392	− 12.9, 2.4, 6.8	− 12, 0, 4	Lentiform Nucleus Gray Matter	9.55
Cluster 5	160	− 23.9, − 7.9, 12.1	− 24, − 8, 12	Putamen	9.02
Cluster 6	152	20.2, − 11.8, 8.1	20, − 12, 8	Thalamus	8.95
Cluster 7	136	12.2, 0.1, 4.1	12, 0, 4	Thalamus	8.86
Cluster 8	80	− 27.6, − 3.6, 4.4	− 28, − 4, 4	Putamen	8.80

Patients					
N/A					

Language					
Controls					
Cluster 1	1032	− 16.5, 11.6, 3.7	− 18, 10, 4	Putamen	12.44
			− 16, 20, − 2	Caudate (head)	9.00
			− 10, 10, 10	Caudate (body)	7.78
Cluster 2	352	− 10.5, − 2.8, 17.7	− 10, 0, 16	Caudate (body)	9.27
			− 12, − 8, 20	Caudate (body)	7.88
Cluster 3	152	− 20.2, 9.6, − 7.4	− 20, 10, − 8	Putamen	8.80
Cluster 4	136	28.3, − 14.1, − 7.9	28, − 14, − 8	Lateral Globus Pallidus	8.75
Cluster 5	136	− 0.01, 12.2, 1.9	0, 12, 2	Caudate (body)	8.77
Cluster 6	128	22.2, 13.6, − 7.5	22, 14, − 8	Putamen	8.69
Cluster 7	128	10, 23.4, 4.6	10, 24, 4	Caudate (body)	8.64
Cluster 8	128	− 20.2, − 43.8, 9.7	− 20, − 44, 10	Brodmann area 30	8.72
Cluster 9	120	20.3, 7.7, − 11.3	20, 8, − 12	Putamen	8.66
Cluster 10	120	− 26.3, − 16.1, − 10.4	− 26, − 16, − 10	Lateral Globus Pallidus	8.70
Cluster 11	120	12.3, 13.3, 20.3	12, 14, 20	Brodmann area 33	8.67
Cluster 12	80	− 25.2, 1.8, 1.6	− 26, 2, 2	Putamen	7.58

Patients					
Cluster 1	760	− 22.3, 0, 10.6	− 22, 0, 10	Putamen	9.14
Cluster 2	664	22, 28, 4	22, 28, 4	Caudate (body)	7.96

Motor					
Controls					
Cluster 1	2200	− 21.6, 3.6, − 2.6	− 20, 4, − 4	Putamen	19.48
			− 26, 2, 8	Putamen	9.37
Cluster 2	1424	23.6, 2.4, 7.2	22, − 2, 10	Putamen	13.53
			28, − 2, − 2	Putamen	9.69
Cluster 3	256	− 29.7, − 11.7, − 1.9	− 30, − 12, − 2	Putamen	9.29

Patients					
Cluster 1	3136	22.8, − 1.5, 6.9	24, 2, 10	Putamen	16.49

Reward					
Controls					
Cluster 1	3760	15.1, 6, 3.6	14, 6, 10	Caudate (body)	21.75
			16, 6, − 2	Lateral Globus Pallidus	13.71
			30, 4, 0	Putamen	9.80
			30, 0, 2	Putamen	9.50
Cluster 2	2440	− 13.8, 6.8, − 0.08	− 10, 8, 0	Caudate (head)	18.16
			− 24, 4, − 6	Putamen	13.91
			− 10, 2, 8	Caudate (body)	9.51
			− 6, 4, 16	Caudate (body)	8.74
Cluster 3	2112	3.7, − 21.5, − 12.5	4, − 23, − 10	Red Nucleus	19.83
			8, − 18, − 10	Red Nucleus	17.19
			− 2, − 24, − 18	Red Nucleus	14.47
Cluster 4	64	− 11, − 7, 3	− 12, − 6, 2	Thalamus	8.00

Patients					
Cluster 1	1648	− 13.9, 10.9, − 8.1	− 12, 10, − 6	Caudate (head)	15.71
Cluster 2	816	12.9, 12, − 3.2	12, 12, − 2	Caudate (head)	8.96
			14, 12, − 12	Sub-lobar Gray Matter	8.88
			12, 12, 4	Caudate (body)	8.72
Cluster 3	664	25.6, 6, 9	26, 6, 8	Putamen	11.72
Cluster 4	160	9, 24, 6	10, 24, 6	Caudate (body)	8.41
Cluster 5	160	15, 3, 18	14, 4, 18	Caudate (body)	8.07
Cluster 6	160	15, 0, 27	15, 0, 27	Brodmann area 24	8.06

Working memory					
Controls					
Cluster 1	2344	− 17.6, − 0.2, 11.6	− 18, 0, 10	Putamen	30.11
Cluster 2	1112	− 20.3, 10.1, − 8	− 22, 10, − 10	Putamen	13.47
			− 12, 10, − 4	Lateral Globus Pallidus	8.29
Cluster 3	640	26.2, 5.9, − 8	26, 6, − 8	Putamen	11.13
Cluster 4	496	11.4, 14.4, − 8.3	14, 16, − 6	Caudate (head)	8.55
Cluster 5	160	− 3.9, 10.1, − 2.1	− 4, 10, − 2	Caudate (head)	8.05

Patients					
Cluster 1	848	17.7, 12.3, − 0.4	18, 8, − 2	Putamen	7.51
			18, 16, 0	Caudate (body)	6.58
Cluster 2	416	4.2, 8.2, 3.5	4, 8, 4	Caudate (head)	8.28
Cluster 3	392	− 8.2, 12.4, 3.8	− 8, 12, 4	Caudate (body)	8.28

All tasks combined					
Controls					
Cluster 1	30,992	2, 4.7, 2.4	− 18, 0, 10	Putamen	38.16
			− 22, 6, − 6	Putamen	37.68
			14, 6, 10	Caudate (body)	32.87
			16, 6, − 2	GPe	26.49
			28, 6, 0	Putamen	24.75
			22, − 2, 12	Putamen	22.75
			26, 0, 2	Putamen	22.74
			26, − 2, 8	Putamen	21.07
			12, 12, − 12	Caudate (head)	19.98
			− 8, 10, − 14	Caudate (head)	15.39
			30, − 12, − 6	Putamen	14.38
			− 30, − 12, − 2	Putamen	10.07
Cluster 2	1464	3.8, − 21.5, − 124	4, − 22, − 10	Red Nucleus	19.83
			8, − 18, − 10	Red Nucleus	17.20
			− 2, − 24, − 18	Red Nucleus	14.47

Patients					
Cluster 1	8960	21, 2, 3.9	24, 4, 10	Putamen	27.34
			24, − 4, 10	Putamen	17.99
			28, − 2, − 2	Putamen	16.20
			20, − 6, − 2	GPi	15.89
			16, 10, − 12	Putamen	15.50
			16, 2, 18	Caudate (body)	14.76
			14, 14, − 12	Caudate (head)	14.55
			26, − 16, − 2	GPe	13.54
			14, 12, − 2	Caudate (head)	12.45
			16, 0, 26	Caudate (body)	10.04
Cluster 2	3928	− 15, 10.5, − 3.9	− 14, 12, − 10	Putamen	21.13
			− 12, 10, − 2	Caudate (head)	20.67
			− 18, 12, − 12	Putamen	19.73
			− 20, 10, 8	Putamen	12.10
			− 22, 2, 10	Putamen	10.36
Cluster 3	736	21.8, 27.4, 6.8	22, 28, 8	Caudate (body)	15.12

Across emotion studies, there were several significant clusters of activation in control groups, primarily in the caudate. Overlap in activation during executive function tasks was seen primarily in anterior regions of the putamen, while that for language was seen across the caudate and putamen, but also included areas in the globus pallidus *pars externa* (GPe). Motor activation overlap in controls was also localized to the putamen. Perhaps not surprisingly, reward tasks showed overlapping activation in regions of the brainstem due to areas that were described in the original works as “ventral striatum”, but there were additional overlaps in the caudate, putamen, and GPe. Finally, analysis of working memory tasks across studies demonstrated significant overlap in the caudate head and putamen, as well as one pallidal region (GPe).

**Fig. 2:**
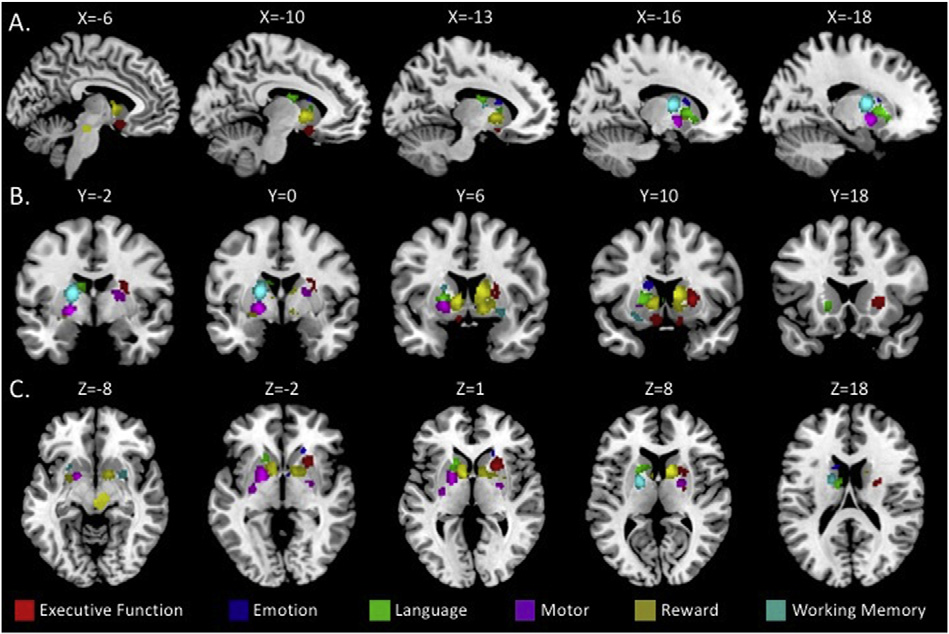
Functional topography of basal ganglia activation. Unique areas of overlap across studies for each task domain, when compared with activation combined across all other tasks, are shown in the sagittal (**A**), coronal (**B**), and axial planes (**C**).

When we contrast individual task domains relative to activity overlap across all other tasks, we begin to see further evidence in support of a functional topography in the basal ganglia of healthy controls, particularly with respect to the specificity of these activation patterns. Detailed coordinates describing these differences within healthy controls are presented in Table 3, and visualized in Fig. 2. Interestingly, when compared to the combined activation of all other tasks investigated here, we found distinct activation foci across all behavioral domains of interest. With respect to executive function, the largest distinct area of overlap was in the right rostral putamen, though there was also a large cluster of overlap in the caudate head. Notably, these areas of overlap are in the rostral aspects of these structures, areas which are connected to medial prefrontal (MPFC) and orbital frontal cortex (OFC), as mapped by Draganski et al. (2008). Contrasting activation across emotion tasks with all other task domains revealed two significant clusters in the caudate head, and one in the caudate body, analogous to regions connected to the MPFC and OFC (Draganski et al., 2008). The region in the caudate body is located such that it overlaps with areas more likely to be connected with dorsolateral prefrontal cortex (DLPFC).

**Table 3:** Distinct basal ganglia by individual task domain in control individuals only. For each task, activation patterns across studies for one domain were contrasted with the activation concatenated for every other domain of interest. Thus, this represents distinct activation for a given task.

Cluster	Cluster size (mm^3^)	Weighted center (x,y,z)	Local extrema (x,y,z)	Location	ALE value (× 10^− 3^)
Executive function					
Cluster 1	2208	24.3, 8.9, 6.3	22, 12, 2	Putamen	3.72
			28, − 4, 18	Putamen	2.38
			22, 8, 14	Caudate (body)	2.17
			24, − 8, 10	Putamen	1.96
			28, 2, 20	Claustrum	1.76
Cluster 2	664	− 7.6, 11, − 13.7	− 6, 8, − 10	Caudate (head)	1.85
			− 8, 15, − 17	Caudate/Gray Matter	1.82
Cluster 3	232	12.4, 11.1, − 15.3	12, 10, − 14	Caudate/Gray Matter	1.89

Emotion					
Cluster 1	288	− 14, 11.2, 15.2	− 14.2, 12.5, 15.4	Caudate (body)	1.81
Cluster 2	168	19.6, 25.4, − 3.4	18, 26, − 6	Caudate (head)	2.79
Cluster 3	64	5.3, 3.2, − 3.2	6, 4, − 4	Caudate (head)	1.71

Language					
Cluster 1	1256	− 17.6, 11.9, 3.2	20, 12, 4	Putamen	3.29
Cluster 2	360	− 10.9, − 4.7, 18.4	− 10, − 4, 20	Caudate (body)	2.40
			− 10, − 8, 18	Caudate (body)	2.36
Cluster 3	72	− 2, 12.2, 3.1	− 4, 14, 2	Caudate (body)	1.84

Motor					
Cluster 1	1532	− 20.2, 2.4, − 3.1	− 18, − 2, − 4	Globus Pallidus *pars externa*	3.35
			− 18, 3, − 5	Globus Pallidus *pars externa*	3.12
Cluster 2	904	22.1, − 4.1, 7.7	24, − 6, 0	Globus Pallidus *pars externa*	2.44
			22, − 4, 8	Globus Pallidus *pars externa*	2.36
Cluster 3	280	− 29.6, − 11.4, − 1.7	− 26, − 10, − 2	Globus Pallidus *pars externa*	2.26

Reward					
Cluster 1	3240	12.8, 6.7, 3.9	8, 8, 10	Caudate (body)	3.54
			11, 12, 9	Caudate (body)	3.19
			14, 6, − 6	Globus Pallidus *pars externa*	2.77
Cluster 2	2112	3.6, − 21.4, − 12.6	4, − 14, − 10	Midbrain (Mammilary Body)	3.04
			0, − 20, − 9	Midbrain (Red Nucleus)	3.01
			− 4, − 20, − 14	Midbrain (Red Nucleus)	2.99
			8, − 16, − 9	Midbrain (Substantia Nigra)	2.88
			7.6, − 18, − 14.8	Midbrain (Red Nucleus)	2.83
			1.2, − 24.5, − 15	–	2.61
Cluster 3	1416	− 9.7, 8.1, 0.4	− 9, 6, 1	Caudate (Head)	3.09
Cluster 4	152	− 25.2, 1.2, − 8.6	− 26, 0, − 10	Putamen	1.72
Cluster 5	56	31.4, 2, 2	32, 2, 2	Putamen	1.95

Working memory					
Cluster 1	1664	− 18.1, − 1.2, 11.7	− 17.9, − 1.3, 12.5	Thalamus	3.71
Cluster 2	320	27, 5.2, − 8.5	26, 8, − 8	Putamen	1.85
			28, 8, − 12	Putamen	1.85
Cluster 3	264	− 24.2, 11.3, − 11.1	− 22, 12, − 14	Putamen	1.95
			− 26, 13, − 11	Putamen	1.94

Distinct activation overlap associated with language task was localized to the left hemisphere, and relatively rostral. Indeed, the peak activation area in the putamen is quite similar to what we found with respect to executive function (see Table 3). However, there was also overlap in caudal regions of the caudate body, where connectivity is strongest with sensorimotor regions (Draganski et al., 2008). Motor task overlap across studies was distinct in the globus pallidus *pars exerna* (GPe). The largest cluster was localized to the left hemisphere, consistent with the laterality of motor connections, and the predominant focus on right-handed individuals in brain imaging research, though there was also a cluster in the right hemisphere as well. Within the GPe, the activation overlap was localized across much of the structure, though notably, Cluster 3 is in the caudal aspects where connectivity is with motor cortical regions of the brain (Draganski et al., 2008).

Reward tasks showed the largest number of independent clusters of overlap when compared to the other task domains in healthy controls (5 clusters, Table 3). Most notably, was the distinct activation in the midbrain, including the substantia nigra. However, there was also distinct overlap in the caudate head, rostral aspects of the GPe, and the caudate body. Finally, across investigations of working memory, in control subjects we see a distinct region of overlap in the thalamus, which extends into the neighboring regions of the basal ganglia. Specific to the basal ganglia however, there is overlap in the bilateral putamen. These clusters are in more ventral regions of the putamen, that likely to be connected with dorsolateral prefrontal cortical regions (Draganski et al., 2008).

### 3.2 Task activation overlap in patients with schizophrenia

In patients we did not find any significant overlap in basal ganglia activation in the emotion and executive function task domains. Analysis of language tasks revealed activation overlap across studies in both the putamen and caudate, while that of motor tasks revealed a region of significant overlap in the putamen. Thus, results of the language and motor task analyses were comparable to controls, when we look at general patterns of activation. Across reward tasks there was significant activation overlap in the caudate and putamen in patients with schizophrenia, but unlike in controls we did not see any clusters in the ventral striatum/midbrain. Finally, in patients with schizophrenia, across studies of working memory, there were overlaps in activation in both the caudate (head and body) and putamen. However, it is important to note that there were fewer activation foci in the patients with schizophrenia generally (this is further discussed in the next section, below) which likely contributed to the fewer areas of activation overlap seen in this group. Notably, the patterns of activation across tasks are relatively complicated. However, for our purposes here, our primary interest is in the topography of these overlaps in controls, and the group differences in activation overlap when comparing controls with patients with schizophrenia. As such, we refer readers to Table 2 and Fig. 1 for more detailed information about these areas of overlap.

### 3.3 Group differences in task activation

As noted above, there were several task domains without any significant activation overlaps in the patients with schizophrenia. In looking at the number of foci per investigation (Table 1) there were fewer foci in the patient group, suggestive of overall decreased activation or a lack of recruitment of the basal ganglia relative to healthy controls across the studies surveyed and included here. To investigate this empirically, we computed the percentage of the total number of studies that had activation in the schizophrenia and control groups respectively (Table 4) and used a *t*-test to compare the two groups across task domains. Patients with schizophrenia had activation in 41.21% of the included investigations, while controls had basal ganglia activation foci in 87.27% of investigations, and this was a statistically significant difference (t_(10)_ = − 3.098, p = 0.011).

**Table 4:** Percentage of studies showing basal ganglia activation in each group by task domain. The percentages below represent the studies that include both patient and control participants. *Domains wherein one additional study was included that investigated only patients with schizophrenia. Healthy controls were not included in the study, and thus are not included in the percentages below.

Task domain (total studies)	Patients	Controls
Emotion (7)*	0%	100%
Executive function (7)	14.28%	100%
Language (5)	80%	60%
Motor (6)	66.67%	100%
Reward (4)*	50%	100%
Working memory (11)	36.36%	63.63%
Average across domains	41.21%	87.27%

**Fig. 3:**
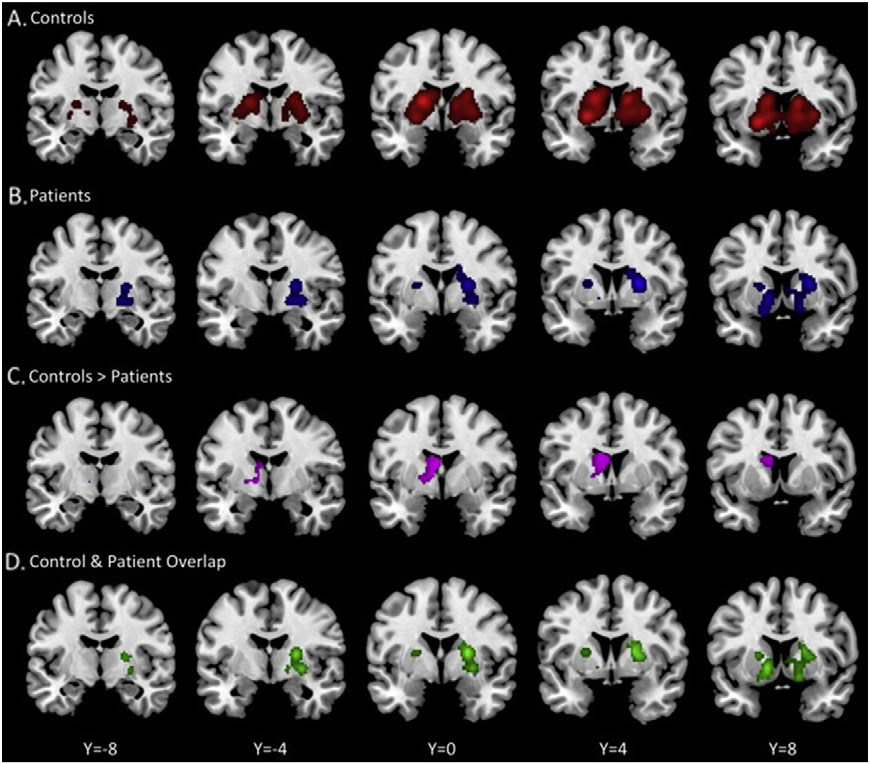
ALE results maps when collapsing foci across all task domains presented on coronal slices for the controls (**A**) and the patients with schizophrenia (**B**). Areas where activation was significantly higher in the controls relative to the patients (**C**) and areas where there was significant overlap across the two groups (**D**) are also pictured. There were no areas that showed significantly greater activation across studies in the patients with schizophrenia relative to the controls.

To further support this overall general decrease in activation within the patient group, we computed a group comparison collapsing across all tasks. Individual group patterns of overlap collapsing across all task domains, and the results of the group comparison analysis are presented in Table 5. Most importantly, this group comparison when collapsing across all task domains provides further evidence for generalized under-recruitment of the basal ganglia in patients with schizophrenia when compared with controls. Notably, as described above, the ALE analyses control for the number of participants in a given study, and as such the impact of differences in the number of participants across groups is minimized. While there were several clusters of significant overlap in activation across tasks when looking at the conjunction of both groups, there were no areas of greater activation when looking at across study overlap in the patient group relative to controls. There was however significantly greater activation overlap in the control group in a large cluster in the caudate, extending into the thalamus when compared to the patient group (Fig. 3).

**Table 5:** Basal ganglia activation overlap comparisons for each individual task domain, as well as collapsing across all task domains. We present significant group differences and conjunctions for each task domain and collapsed across all tasks. Because there were no significant foci in the emotion and executive function/attention domains, group comparisons were not computed. In addition, there were no significant group differences or overlaps when we computed the analyses of working memory foci, and as such it has not been included in the table. When collapsing across all task domains, there were no clusters where activation was greater in the patients with schizophrenia as compared to the controls. GPe: globus pallidus pars externa; GPi: globus pallidus pars interna.

Cluster	Cluster size (mm^3^)	Weighted center (x, y, z)	Local extrema (x, y, z)	Location	ALE value (× 10^− 3^)
Language					
Controls > patients					
Cluster 1	320	− 17.3, 11.7, 2	− 16, 6, 2	Putamen	2562.24
			− 17.4, 12.5, 2.1	Putamen	2467.66
Cluster 2	64	− 11, 10.5, 8.5	− 10.4, 10.4, 9.2	Caudate (body)	2489.29

Motor					
Group overlap (conjunction)					
Cluster 1	912	22.8, − 2.1, 9.4	22, 0, 10	Putamen	12.65
			24, − 4, − 2	GPe	6.10
Cluster 2	8	26, − 6, − 2	26, − 6, − 2	GPe	4.96

Reward					
Group overlap (conjunction)					
Cluster 1	608	− 12.8, 9.5, − 3.7	− 12, 8, − 4	GPe	13.83
Cluster 2	280	12.1, 11, 2.8	12, 12, 4	Caudate (body)	8.72
			12, 10, − 2	Caudate (head)	7.90
Cluster 3	16	15, 4, 16	14, 4, 16	Caudate (body)	7.10

All tasks combined					
Controls > patients					
Cluster 1	1344	− 8.6, 2.1, 14.1	− 6, 0, 10	Thalamus	3352.80
			− 10, 20, 10	Caudate (body)	3090.23
			− 6.8, 3.2, 20.4	Caudate (body)	2807.03
			− 16, 0, 2	GPe	2530.19

Group overlap (conjunction)					
Cluster 1	6608	20.9, 3.4, 2.9	24, 0, 12	Putamen	20.19
			24, − 4, 10	Putamen	17.99
			28, − 2, − 2	Putamen	16.20
			14, 10, − 12	Caudate (head)	14.62
			14, 14, − 12	Caudate (head)	14.55
			14, 12, − 2	Caudate (head)	12.45
			28, − 14, − 4	GPe	11.75
			20, − 2, − 2	GPe	11.30
Cluster 2	2984	− 14.7, 9.7, − 1.5	− 12, 10, − 2	Caudate (head)	20.67
			− 14, 10, − 8	Putamen	18.59
			− 18, 12, − 10	Putamen	18.43
			− 10, 12, − 12	Caudate (head)	12.88
			− 18, 12, 6	Putamen	12.05
			− 20, 8, 8	Putamen	12.00
			− 22, 2, 10	Putamen	10.37

The results of the group comparisons by individual task are also presented in Table 5. Since there were no significant areas of overlap in the patients with schizophrenia for both the emotion and executive function domains, we were unable to calculate any group differences or overlaps between the two groups for these domains. With respect to language, there were no regions of overlap between the two groups; however, controls showed significantly larger regions of activation across investigations in the caudate and putamen during language-related tasks. There were no regions where there was greater activation across tasks in the patient group when compared to controls. In the motor domain, there were no group differences in either direction, though there was significant overlap in the putamen and the GPe. Similarly, with reward, we only found significant areas of overlap in the GPe and caudate. However, though the controls showed significant patterns of activation across studies in the ventral striatum and midbrain, this was not significantly higher in the group comparison, perhaps due to the lower power for this analysis. Finally, there were no differences in working memory, nor were there any regions that were significant in our conjunction analysis.

## 4 Discussion

Using ALE meta-analysis we investigated basal ganglia activation patterns across task domains in patients with schizophrenia in comparison with healthy controls. First we demonstrated a functional activation topography in healthy controls consistent with the literature investigating parallel loops with the cerebral cortex (Draganski et al., 2008, Di Martino et al., 2008). Looking across cognitive, motor, and affective task domains, there was a striking general pattern of decreased basal ganglia recruitment in the patient group. Together, these findings provide important new insight into the functional topography of the basal ganglia and basal ganglia function in schizophrenia. We first discuss the patterns seen in healthy controls to provide appropriate context for discussion of the patient findings, particularly in the context of the dopamine hypothesis.

### 4.1 Activation overlap in healthy individuals and basal ganglia circuits

In healthy adults, there are distinct circuits of the basal ganglia connecting these subcortical nuclei to different regions of the prefrontal cortex. Broadly, these circuits are defined by limbic, prefrontal, and motor/occulo-motor connections (Alexander and Crutcher, 1990, Alexander et al., 1986). In general, the task domains we looked at group roughly into the primary parallel loops of the basal ganglia circuits, though we did not investigate occulomotor tasks. Motor activation in healthy controls is localized in the putamen when looking at motor activation on its own (Di Martino et al., 2008, Postuma and Dagher, 2006). Generally, this finding is highly consistent with our understanding of the role of the putamen in motor function. Relative to the activation overlap for all other tasks, we found activation overlap clusters specific to motor tasks in the GPe, in regions associated with basal ganglia-motor loops (Draganski et al., 2008). While this specific activation in the GPe is not surprising given the connectivity pattern of this region, this novel finding is of great interest, given that the putamen is often associated with motor function. The role of the basal ganglia in language is less clear, but it has been suggested that the putamen may be involved in initiating phonological representations important for language production and processing (Booth et al., 2007) and in the sequencing of linguistic information (Chan et al., 2013). As evidenced by the included foci described in Table 2, it is clear that the basal ganglia are activated across a variety of language processing tasks. The broad pattern of activation seen across language studies may be due to initiation and sequencing with respect to language in ways that are analogous to what occurs during motor processing and sequencing (Booth et al., 2007, Chan et al., 2013). Activation overlap specific to language however was focused on the putamen and caudate in the left hemisphere, consistent with the lateralization of language processes. The specific putamen region was quite rostral, and consistent with areas connected to the prefrontal cortex (Draganski et al., 2008) while the areas of overlap in the caudate are in regions associated with sensorimotor function. The rostral overlap and resulting prefrontal overlap may be due to the highly cognitive nature of many of the tasks included in this investigation (Table 1), while the areas associated with sensorimotor networks may reflect the motor aspects related to speech and language, though these tasks were non-vocal.

Tapping into the pre-frontal/associative loop are the executive function/attention and working memory domains. Activation overlap across tasks investigating executive function and attention was largely in the putamen, but localized to the anterior regions (Fig. 1A). When we tested the specificity of the overlap across executive function tasks, they were indeed localized to the rostral putamen, but also the caudate head. This anterior region of the putamen and the caudate head are both associated with the prefrontal cortex, specifically MPFC and OFC (Draganski et al., 2008), the latter of which is heavily implicated in executive function (Orr and Banich, 2014, Orr et al., 2015). The implication of the putamen and caudate head in this functional domain is consistent with the connectivity patterns of this region (Draganski et al., 2008, Di Martino et al., 2008). Working memory overlap was seen in the caudate head and putamen, though activation specific to working memory tasks was localized to the thalamus and putamen. Interestingly, and importantly, the unique regions of working memory activation overlap were in more caudal regions of the putamen then those associated with executive function (Fig. 2), and these areas are associated with dorsolateral prefrontal cortex, which has been heavily implicated in working memory processes (e.g., Jonides et al., 1993, Reuter-Lorenz et al., 2000).

Finally, both emotion processing and reward tasks allow us to investigate the basal ganglia in light of the limbic loop. Consistent with the animal and human literature on reward processing (eg., Schultz et al., 1997, Schultz et al., 2000, Schultz et al., 1998, Sesack and Grace, 2010, Tanaka et al., 2016), we saw overlap during reward tasks in the ventral striatum and regions of the midbrain analogous to the substantia nigra. Our investigation of distinct activation during reward processing also revealed midbrain regions that were unique to the domain, in addition to rostral regions in the GPe and caudate head. These more rostral regions of overlap are consistent with the fronto-striatal circuitry, particularly in the OFC thought to underlie reward processing as investigated in non-human primate models (Schultz et al., 2000). Emotion processing tasks were primarily localized to the head of the caudate, though these regions extended into ventral striatal regions, consistent with the limbic loops of the basal ganglia. The clusters in the rostral caudate are analogous to regions connected to the MPFC and OFC (Draganski et al., 2008). Indeed, these frontal regions have been implicated in emotion regulation and the cognitive control of emotion (reviewed in Etkin et al., 2011, Ochsner and Gross, 2005).

Together, these findings provide key new insights into the functional topography of the basal ganglia and to our knowledge represent the first meta-analytic investigation of basal ganglia function of its kind. This builds upon our understanding of cortico-striatal loops (eg., Alexander and Crutcher, 1990, Alexander et al., 1986, Hoover and Strick, 1993) and provides an important point of comparison for investigations of psychopathology where the basal ganglia are implicated. Most critically, with our analysis of the *unique* areas of overlap with each task domain, we were able to produce a map of functional topography in the basal ganglia that is highly consistent with the connectivity patterns of the structure (Draganski et al., 2008, Di Martino et al., 2008). We suggest that activation across basal ganglia nuclei and different task domains is due to the processing in these regions being conducted on distinct input from different cortical regions, and are subsequently relaying the output of these neural computations back to the cortical targets.

### 4.2 Activation overlap differences in patients with schizophrenia

Most notably, we demonstrated that there are generally fewer areas of activation overlap in patients with schizophrenia. As noted in our results, when we compare the percentage of studies that had basal ganglia activation foci in the patient group, this was significantly smaller than in the controls. When looking at individual task domains, there are no domains in which there was more activation seen in patients relative to controls, and this is consistent with our results seen when we collapse across all task types. Though there are several large areas of overlap suggesting that the patients are recruiting these nuclei to some degree, patients with schizophrenia are not activating the basal ganglia across task domains and studies to the same extent as healthy controls. Together, this suggests broad basal ganglia dysfunction across studies and samples of patients with schizophrenia, and to our knowledge this is the first large-scale analysis of basal ganglia activation patterns in patients with schizophrenia, making these findings especially novel.

### 4.3 Implications for our understanding of disease

In light of the proposed contributions of the basal ganglia to the etiology of schizophrenia, these patterns are especially revealing. Dopaminergic function has been heavily implicated in schizophrenia, as is underscored in the dopamine hypothesis (Howes and Kapur, 2009). It is also consistent with earlier suggestions that fronto-striatal function is altered in patients with schizophrenia (Robbins, 1990). However, it is notable that the typical pattern of dopaminergic progression with the development of schizophrenia suggests increases in striatal dopamine synthesis (Howes et al., 2011a, Howes et al., 2011b) while our results here demonstrate an overall decrease in basal ganglia activation across task domains. In patients with Huntington's Disease, as well as in those in the pre-clinical stages of disease, there are also patterns of decreased brain activation relative to controls, including activation in the basal ganglia (Kim et al., 2004, Wolf et al., 2007, Zimbelman et al., 2007), though this is a disease characterized by hyperkinetic movements and an excess of dopamine. Thus, our general findings of decreased basal ganglia activity even in the presence of an increase in striatal dopamine are consistent with those seen in Huntington's Disease.

Schizophrenia is characterized by heterogeneous symptom profiles featuring both positive and negative symptoms (Andreasen and Olsen, 1982), in addition to cognitive dysfunction (eg., Barch and Smith, 2008, Elvevag and Goldberg, 2000, Heinrichs and Zakzanis, 1998, Ranganath et al., 2008) and motor abnormalities (eg., Bernard and Mittal, 2014, Bernard et al., 2014, Caligiuri and Lohr, 1994, Mittal and Walker, 2013, Mittal et al., 2007, Morrens et al., 2014, Walther and Strik, 2012). While the dopamine hypothesis and elevated striatal dopamine has been linked to the positive psychotic symptoms of the disease (Howes and Kapur, 2009) and likely contributes to some of the motor abnormalities seen in schizophrenia (Walther and Strik, 2012), this overall pattern may be important for our understanding of negative symptoms and cognitive dysfunction as well. While recent work has linked negative symptoms to cerebellar-mediated behaviors (Bernard et al., 2014, Dean et al., 2015), the basal ganglia may also be an important locus for our understanding of this key symptom domain. Crucially, negative symptoms have been linked to hypofrontality in patients with schizophrenia (Potkin et al., 2002, Wolkin et al., 1992) and the limbic loop of the basal ganglia is of particular interest. Indeed, in a review of reward processing in schizophrenia, it was suggested that aberrant cortical-striatal interactions with respect to this domain may be especially important for our understanding of the negative symptoms present in the disease (Strauss et al., 2014). Negative symptoms include apathy and a general lack of motivation, which is consistent with the dampened basal ganglia activation found here across studies investigating reward processing and emotion. Indeed, unlike in controls, there were no significant foci of activation in the basal ganglia across emotion tasks. Furthermore, in these investigations of hypofrontality it is notable that regional blood flow in the basal ganglia is one of the regions correlated with negative symptom severity (Potkin et al., 2002), while Juckel et al. (2006)) found that functional activation in the ventral striatum during reward processing was correlated with negative symptom severity in patients with schizophrenia. Together, these findings and our evidence for decreased recruitment of the basal ganglia in patients with schizophrenia provide support for the importance of this region, particularly for our understanding of both the positive and negative symptom domains.

With respect to cognition, as we demonstrated here, there is robust activation in the basal ganglia across studies and cognitive task domains. The general lack of activation in patients with schizophrenia, particularly relative to controls in these domains suggest that the basal ganglia may be contributing to the wide array of cognitive deficits experienced by these patients. Most likely, this is mediated by the connectivity with the prefrontal cortex (eg., Alexander and Crutcher, 1990, Di Martino et al., 2008, Draganski et al., 2008, Hoover and Strick, 1993, Middleton and Strick, 2002). Thus, the marked differences and general decreases seen in basal ganglia activation in patients with schizophrenia relative to controls suggest that basal ganglia deficits are present in a variety of domains, and may have broader reaching impacts for symptoms and function in this population. Indeed, studies have indicated that basal ganglia activation is related to symptom severity in patients with schizophrenia (Gradin et al., 2013, Simon et al., 2010).

### 4.4 Limitations

While our investigation here provides key new insights into basal ganglia function in patients with schizophrenia, there are several limitations to consider. First, this patient population is heterogeneous. Participants across the included studies were often on medication of different types, had comorbidities, and varied with respect to time since diagnosis. Medication in particular has wide ranging effects and may influence motor function, though cognition, emotion and reward processing may all be impacted as well. Importantly, we were unable to incorporate medication status in our analyses. Information reported varied across studies, and currently, the ALE methods included here do not allow for the inclusion of additional variables such as this. Similarly, as previously noted, there is substantial evidence to suggest that the basal ganglia are smaller in patients with schizophrenia. This heterogeneity may also contribute to the activation patterns and group differences found here and we were unable to account for volumetric differences in our analyses. Second, the way in which we determined studies for inclusion was specific to the basal ganglia. We only included studies that showed basal ganglia activation. There are many additional studies that investigate similar task domains, but may not have shown basal ganglia activation in patients or controls. As such, though we have collapsed across numerous investigations, this analysis does not take into account every neuroimaging study of patients with schizophrenia, as is standard with this type of investigation (e.g., Bernard and Mittal, 2014). Thus, our results are only indicative of those investigations that showed basal ganglia activation, and our interpretations should be considered with this in mind. Further, we focused solely on the basal ganglia themselves and did not investigate concomitant cortical activation. Future work would benefit from taking this into account.

We surveyed a significant proportion of the literature, and searched broadly across all studies in Pubmed using neuroimaging in patients with schizophrenia, but we ultimately were only able to focus on studies that showed activation in the basal ganglia in one group. While an investigation of the consistency of basal ganglia activation within a given domain would be quite interesting, it is beyond the scope of this investigation. Relatedly, our functional topography in healthy controls is somewhat limited given that this is a smaller sampling of studies, limited to cases where healthy individuals were serving as controls for patients with schizophrenia. Thus, our healthy control topography is that which is present in this limited sample. In our *t*-test comparison of control and patient groups investigating the relative proportion of studies showing the basal ganglia, it is important to note that this does not account for relative sample size (unlike our ALE analysis). The general lack of activation in some domains and studies contributing to these group differences may be driven by sample size differences and statistical power. With that said, this is somewhat unlikely, given that across all 40 studies included in our analyses here, there were more patients than controls (n = 707 patients; n = 583 controls). Finally, we must consider the contributions of task demands. While we did not assess or quantify behavioral differences in the studies we included as we were focused solely on activation differences, it is notable that these investigations were designed for clinical populations. With that in mind, task demands were likely not optimal for the control group, to ensure that the patients are able to effectively complete the task of interest. This may as a result be impacting our findings. If anything, we suggest that if the tasks were better optimized for healthy controls, the differences with patients may be even more striking.

## 5 Conclusions

Using state-of-the-art neuroimaging meta-analysis methods we investigated basal ganglia activation across task domains (motor, cognitive, and affective) in studies of patients with schizophrenia. Looking at the healthy control data only, we provide a functional topography of basal ganglia activation consistent with the parallel loops seen in the cortex. Crucially, when looking at patients with schizophrenia, we found an overall decrease in basal ganglia activation across all domains, and in specific task domains as well. Indeed, in the emotion and executive function/attention domains there were no significant regions of activation overlap across studies, while this was present for healthy controls. Given the parallel motor/occulo-motor, frontal, and limbic loops between the basal ganglia and cortex, we suggest that the basal ganglia deficits shown here may contribute to both negative symptoms and cognitive dysfunction in patients with schizophrenia, in addition to the positive symptoms and motor dysfunction associated with the dopamine hypothesis.

## Authorship

J.A.B. and V.A.M. developed the study concept and designed the meta-analytic analysis plan. Data collection and preparation of the results was completed by J.AB., C.E.R, R.E.N., and J.R.M.G. Interpretation of the results and drafting of the manuscript was completed by J.A.B. and V.A.M. with critical revisions provided by C.E.R., R.E.N., and J.R.M.G. All authors approved the final version of the paper for submission.
